# It is not in the details: Self-related shapes are rapidly classified but their features are not better remembered

**DOI:** 10.3758/s13421-019-00924-6

**Published:** 2019-03-29

**Authors:** Merryn D. Constable, Jason Rajsic, Timothy N. Welsh, Jay Pratt

**Affiliations:** 1 Department of Cognitive Science, Central European University, Budapest, Hungary; 2 Faculty of Kinesiology and Physical Education, University of Toronto, Toronto, Canada; 3 Department of Psychology, University of Toronto, Toronto, Canada; 4 Department of Psychology, Vanderbilt University, Nashville, TN USA

**Keywords:** Self-referential processing, Self-representations, Memory, Ownership, Self-prioritization

## Abstract

Self-prioritization is a robust phenomenon whereby judgments concerning self-representational stimuli are faster than judgments toward other stimuli. The present paper examines if and how self-prioritization causes more vivid short-term memories for self-related objects by giving geometric shapes arbitrary identities (self, mother, stranger). In Experiment 1 participants were presented with an array of the three shapes and required to retain the location and color of each in memory. Participants were then probed regarding the identity of one of the shapes and subsequently asked to indicate the color of the probed shape or an unprobed shape on a color wheel. Results indicated no benefit for self-stimuli in either response time for the identification probe or for color fidelity in memory. Yet, a cuing benefit was observed such that the cued stimulus in the identity probe did have higher fidelity within memory. Experiments 2 and 3 reduced the cognitive load by only requiring that participants process the identity and color of one shape at a time. For Experiment 2, the identity probe was memory-based, whereas the stimulus was presented alongside the identity probe for Experiment 3. Results demonstrated a robust self-prioritization effect: self-related shapes were classified faster than non-self-shapes, but this self-advantage did not lead to an increase in the fidelity of memory for self-related shapes’ colors. Overall, these results suggest that self-prioritization effects may be restricted to an improvement in the ability to recognize that the self-representational stimulus is present without devoting more perceptual and short-term memory resources to such stimuli.

## Introduction

There has been a recent boom in research regarding self-representational stimuli, with a particular emphasis on attention (see Humphreys & Sui, 2015). The present research centers on processing benefits associated with stimuli that have been arbitrarily assigned to the self, and examines if and how such processing benefits potentially propagate working memory. More specifically, does self-prioritization stop at the rapid identification of self-related objects or does it also produce cognitive effects that result in increased uptake and storage of information related to the self?

Much of the early research demonstrating enhancements to the cognitive processing of self-related stimuli centered on one’s own name (e.g., Arnell, Shapiro, & Sorensen, 1999; Moray, 1959; Shapiro, Caldwell, & Sorensen, 1997; Wood & Cowan, 1995), own face (e.g., Brédart, Delchambre, & Laureys, 2006; Sui, Zhu, & Han, 2006) or virtual items designated as self-owned (e.g., Cunningham, Turk, MacDonald, & Macrae, 2008). These studies have generally pointed to self-predilections in attention or memory as well as judgment and decision making (e.g., Beggan, 1992; Constable, Welsh, Huffman, & Pratt, 2019). There is also evidence that self-predilections in the cognitive system may operate on perceptual- (faces: Ma & Han, 2010; Sui & Han, 2007; self-referential stimuli: Truong, Roberts, & Todd, 2017) and action-related processes (Constable, Kritikos, & Bayliss, 2011; Constable, Kritikos, Lipp, & Bayliss, 2014; Constable et al., 2016).

An elegant experimental procedure used by Sui, He, and Humphreys (2012) pares down ecologically-based self-stimuli to create an arbitrary self-association. Work that uses this procedure provides support for the notion of automaticity in self-based processing (Sui, Sun, Peng, & Humphreys, 2014) without the typical confounds that are present in ecological forms of self-relevance (e.g., familiarity). Participants are told, for example, that they are represented by a circle, their friend (or mother) is represented by a square, and a stranger is represented by a triangle. This rapid induction results in a strong self-association (Wang, Humphreys, & Sui, 2016) with robust processing advantages when participants are asked to judge whether a shape and a label pairing match or not. Such self-association tasks have been extended from shape-based stimuli to avatars (Woźniak, Kourtis, & Knoblich, 2018), color-coded avatars (Sun, Fuentes, Humphreys, & Sui, 2016), and movements (Frings & Wentura, 2014).

Following from the aforementioned research, Humphreys and Sui (2015) provide a framework that is primarily situated within the attentional domain aimed at explaining self-representational effects (the Self Attention Network). The framework is based on the notion that the individual’s self-representation is continuously activated and is thus rapidly triggered by the presence of a self-representational stimulus. This “chronically activated self” interacts with bottom-up orienting processes to generate a self-advantage in attention. Thus, the self-face advantage, for example, could be explained by the rapid engagement of bottom-up orienting processes stemming from a chronically activated self-schema in relation to the participant observing their own face. Top-down attentional control mechanisms may also interact with bottom-up orienting in a facilitatory or inhibitory capacity. Importantly though, self-prioritization effects cannot be abolished through low prior expectancies for such stimuli (Sui et al., 2014). In other words, the theory surrounding the SAN states that self-related attentional processing is in some way special, which in turn predicts that self-related stimuli should show advantages in the amount of information that is incidentally encoded regarding co-occurring features of the stimuli.

What are the consequences of such a bias to self-related stimuli? If it is true that a self-representational stimulus receives attentional priority, then the self-representational stimulus may not simply be noticed more quickly, but the stimulus may also receive enhanced perceptual processing. Bottom-up attention has been shown to improve judgments of contrast (Carrasco, Ling, & Read, 2004; Lui, Abrams, & Carrasco, 2009), spatial frequency (Gobell & Carrasco, 2005), and even facial attractiveness (Störmer & Alvarez, 2016). The amount of information we store in memory about attended features is also affected by what we attend to (Bays & Husain, 2008). If self-representational stimuli induce a bias in visual attention, then the amount of information that one encodes and stores about their appearance may be greater than that for non-self-relevant objects that are presented concurrently. Indeed, the idea of an attentional mechanism propagating to enhanced encoding for self-representational stimuli has been suggested much earlier (Turk, van Bussel, Brebner et al., 2011; Turk, van Bussel, Waiter, & Macrae, 2011; Turk et al., 2013). It is possible, however, that the observed bias to self-representational stimuli is restricted simply to recognition, with no additional differences in how objects are perceptually processed. That is, self-representational stimuli may simply have faster access to semantic memory structures that allow for the recognition of the fact that a self-representational stimulus is in view without a more detailed representation of the stimulus’ features in memory. We will refer to these two possibilities as the visual attention bias account and the self-recognition account, respectively.

In this paper, we contrasted the visual attention bias and self-recognition accounts of self-relevance effects by asking participants to remember and report the specific colors of self- and non-self-relevant objects while performing a categorization task. Psychophysical studies of memory have successfully modeled memory for the features of visual objects as a combination of whether a feature is remembered at all and how precisely a feature is remembered (see Zhang & Luck, 2008; Bays, Catalao, & Husain, 2009; Ma, Husain, & Bays, 2014). Importantly, attention to particular items has been shown to affect the probability that an item is stored in memory (Murray, Nobre, Clark, Cravo, & Stokes, 2013; Souza, Rerko, Lin, & Oberauer, 2014) and sometimes its precision (Gunseli, van Moorselaar, Meeter, & Olivers, 2015; Rajsic, Ouslis, Wilson, & Pratt, 2017). Given the close connection between attention and memory, we predicted that self-representational stimuli, which show speeded processing (Sui et al., 2012), would also be remembered with either greater probability or precision.

For each experiment, participants were required to associate three shapes with three identities: themselves, their mother, and a stranger. The first experiment presented all three shapes simultaneously and required participants to remember the colors and locations of each. Experiments 2 and 3 involved a less cognitively demanding task in which only one of the shapes was presented per trial. The experimental tasks required participants to make two sequential judgments regarding (1) whether a shape and label pair matched or not (Shape Classification, based on Sui et al., 2012), and (2) the color of the target stimulus (Color Estimation, based on Zhang & Luck, 2008). The first judgment was initially memory-based. That is, the shape was indicated after the shape/s had disappeared (Experiments 1 and 2) except in Experiment 3 where the classification was perceptual in nature in that shape and label simultaneously appeared and remained on screen until a response was made. The color estimation judgment in each of the three experiments was always memory based and the target of the judgment was indicated by an uncolored shape.

In line with previous experiments demonstrating self-prioritization with the shape/label matching task, it was generally expected that response times for shape classification would be faster for self-trials than for mother and stranger trials. Indeed, previous studies have demonstrated just this pattern of response times (Sui et al., 2014). If these faster self-judgments reflect rapid perceptual processing, then they ought to occur only when the self-related shape is visible when being judged. However, if rapid self-processing in this task comes from differential post-perceptual processing, then this self-advantage should occur when the judgment is made on remembered shapes as well.

For color estimation, if self-representational stimuli are more likely to attract and sustain visual attention (SAN, Humphreys & Sui, 2015), then participants’ memory for the color of the self-stimulus should be biased such that they can more often, or more precisely, recall the color of the self-related object (Zhang & Luck, 2008). As such, we predicted that, in all the present experiments, memory for the color of self-representational stimuli would be superior to that of mother and stranger shapes consistent with the visual attention account.

## Experiment 1

On each trial we presented participants with three randomly colored shapes: the self shape, the mother shape, and the stranger shape, and participants were required to remember the location and color of each shape. According to the SAN, the presence of a self-representational stimulus should trigger bottom-up orienting processes. If this is the case, attention would not be evenly distributed over the exposure period and any self-prioritization with regard to attention would ensure that the self-stimulus is encoded in working memory and may receive a benefit from enhanced elaborative encoding (Symons & Johnson, 1997). In this sense, it is expected that when memory is probed regarding the identity of a target stimulus, participants would have shorter response times to respond on Self Trials than on Mother and Stranger Trials. Similarly, when memory for the color of the self-stimulus is tested, it should be reported more precisely and/or exhibit a greater likelihood that its color is present in working memory at the time of the probe (p(Mem), Zhang & Luck, 2008).

### Method

#### Participants

A minimum number of participants was set at 20 and all participants who signed up completed the experiment. In total 36 first-year Psychology students participated in this experiment. All participants provided informed consent before participating and were compensated with course credit. The methods employed were approved by the Office of Research Ethics at the University of Toronto.

#### Stimuli and apparatus

Stimuli were generated and presented using MATLAB by Mathworks (Natick, MA, USA) and the Psychophysics toolbox (Kleiner, Brainard, & Pelli, 2007) on 17-in. CRT monitors on a black background. Participants viewed stimuli from a fixed distance (57 cm) using chin rests. Three shape stimuli were randomly sampled for each participant from a set of six shapes (an equilateral triangle, a circle, a hexagon tilted 45° from vertical, a rhombus, a five-sided star, and a heart). All shapes were drawn such that their outer edges were bounded by a 4° square. Fixation was encouraged using a 0.5° “+” symbol in the center of the screen. During induction, shape stimuli were drawn in white (RGB: [255, 255, 255]) and during the experiment, colors were sampled from a color wheel in L*A*B space, centered on [20, 35] with a radius of 50. On a given trial, the colors of the shapes were sampled from a set of eight colors, chosen to ensure 45° of separation but with a random rotation applied so that all colors were equally likely to appear. RGB values were calculated using MATLAB’s makecform and applycform functions. All responses were collected from a standard USB computer mouse with two input buttons (left and right).

#### Procedure

##### Induction

After being greeted and instructed by the experimenter, participants were asked to input their name, their mother’s name, and a stranger’s name into the computer. If the participant asked about the stranger’s name they were given the further instruction of inserting “a random name that does not belong to someone you know.” Proper nouns were used to avoid possible stimulus asymmetries (see Schäfer, Wentura, & Frings, 2017; Wade & Vickery, 2017). The computer script subsequently assigned these names to be randomly paired with three shapes. Participants were then shown the three shapes on the screen simultaneously, and their corresponding labels were presented below the shape.

##### Training

When ready, participants initiated a test session where they were shown one of the three shapes (randomly sampled) with either its correct label or an incorrect label below it (also randomly sampled; 4.8° below the center of the shape, size 18 Arial font). Participants pressed the left mouse button if they believed that the label was correct for that shape, and the right mouse button if they believed it was incorrect. If an error was made, participants were returned to the instruction screen. Participants were required to get nine of these responses correct in a row before they could move beyond the training phase.

##### Experiment

The experimental task combined a shape classification task (Judgment 1, Sui et al., 2012) with a delayed color estimation task (Judgment 2, Zhang & Luck, 2008). Participants completed 324 trials, divided across six blocks. Each trial began with a 1,000-ms fixation display comprised of a single white “+” centered on a black background. Following the fixation display, all three shapes (each with a different random color, as detailed above) were presented on screen for 500 ms. The shapes were presented centered on the perimeter of on an imaginary circle around fixation, radius 8°, at 45° (below and right of fixation), 180° (above fixation), and 315° (below and left of fixation). The specific location of each object on a given trial was randomized. Following a 1,000-ms blank screen, a label was presented in the center of the screen alongside an arrow, 1.25° in length, that pointed towards one of the three locations where the stimuli had been. Participants then responded with the left mouse button if this label matched the shape that had appeared in that location, and with the right mouse button if the label did not match. If the incorrect response was made, feedback was immediately presented in the center of the screen in the following manner: “Incorrect. The shape was <correct label>”, in red font, for 1,000 ms.

**Fig. 1:**
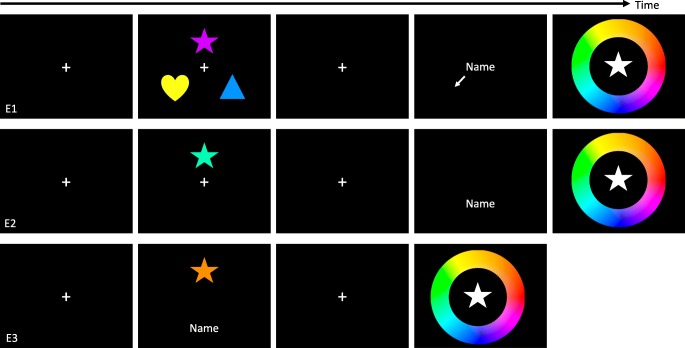
Schematic (not to scale) of the trial structure used for Experiments 1–3. Delay (500 ms) between label response and memory probe is not pictured

Following a correct response to Judgment 1 or an error screen, a blank screen was presented for 500 ms. An object (the shape of one of the objects presented at the start of the trial) appeared in white at the center of the screen. Around this object, a color wheel with a radius of 8.12° and a width of 0.32° that depicted all possible stimulus colors was presented. Participants used the computer mouse to select the color they believed best matched the shape as it had been presented at the beginning of the trial. Once participants moved the mouse 6° or more from the center of the screen, the shape was redrawn in the color corresponding to the angle that subtended between the mouse cursor and the center of the screen. Participants clicked the left mouse button to complete their color choice. After this response, a 1,000-ms delay separated the end of the trial and the start of the next trial (Fig. 1).

#### Data analysis

Participants were retained for analysis if they performed the shape-label judgment with 85% accuracy or greater over the entire experiment (four removed). One participant’s data was also removed because they inserted “Beyonce” as the stranger’s name. Henceforth, the final sample was n = 31. For the shape classification task, mean response times in each condition included only trials with a correct response. For the color estimation task, three measures were calculated, again only including trials where participants classified the shape correctly. One measure was the mean absolute deviation: the average unsigned difference between the reported color and actual shape’s color in a given condition. The other two measures were estimates of the precision of memory (Memory Standard Deviation) and the probability of memory (p(Mem)). Briefly, these two latent memory measures were estimated from report errors by finding the maximum likelihood parameters of a mixture model, which assumes memory error results from several sources: imprecision of color representation (SD) and randomly-distributed responses (1 – p(Mem), resulting from either pure guesses or swap errors). These parameters were estimated separately for each participant and condition using the three-component model (Bays et al., 2009). Note that we focussed our analyses on the probability that an item’s color is remembered and did not focus on the relative contributions of “swap” and guess errors to memory, as color swapping occurred as often for self-stimuli as for non-self-stimuli.

### Results and discussion

Statistical analyses were conducted using JASP using default priors (JASP Team, 2018). We report Bayes factors expressing the probability of the data given the alternative hypothesis relative to the null hypothesis (i.e., values larger than 1 are in favor of the alternative) unless stated otherwise. Recall that the aim of the present experiment was to determine if self-prioritization propagates higher fidelity representations in memory for the self-representational stimulus, as compared to other-representational stimuli. To this end we analyzed response times to the self-classification judgment to determine if self-prioritization occurred. From the color estimation task, we derived three dependent measures that index the probability that the stimulus was in memory and the precision of that representation in memory. We analyzed the data in relation to two factors – Cue Identity and Target Identity – to determine if there were any memory benefits for the self-stimulus as well as if there were any memory benefits to the cued stimulus. The presence of a self-prioritization effect in response times and memory-dependent variables would be consistent with the visual attention hypothesis that self-stimuli enjoy greater attentional prioritization, which then propagates to an enhanced representation in memory.

#### Shape classification judgment

**Fig. 2:**
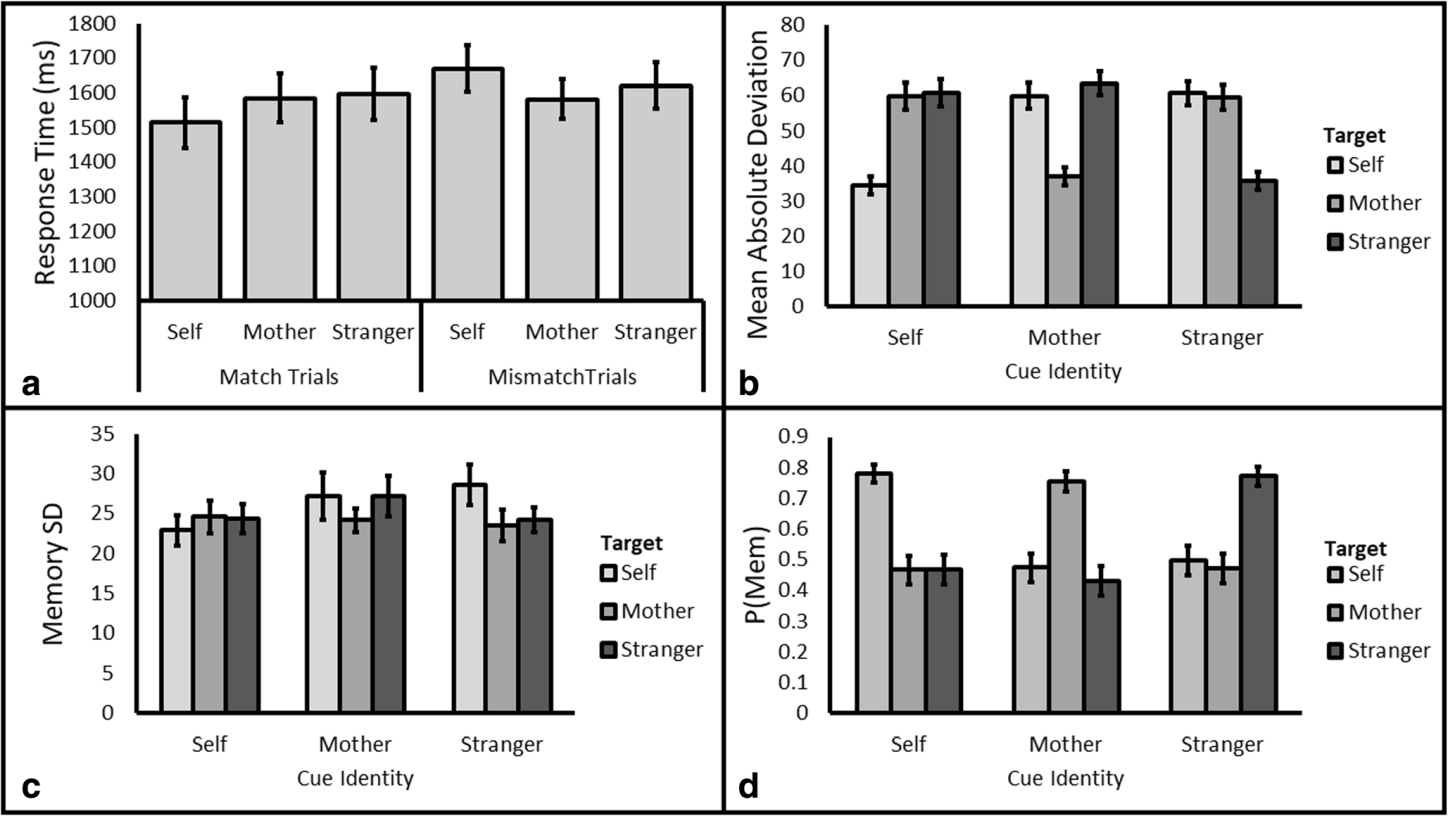
**Panel A:** Response time for match and mismatch trials by Target Identity for Experiment 1. **Remaining panels:** Mean Absolute Deviation (**B**), Memory Standard Deviation (**C**) and P(Mem) (**D**) for Self, Mother, and Stranger shapes by Cue Identity (Self, Mother, and Stranger) for Experiment 1. All error bars are standard error of the mean

Participants were generally accurate in response to the Shape Classification probe (*M* = 93.59%, *SD* = 3.49%), and as such we restrict our analyses to response time. We tested models specifying the factors of Identity (Self/Mother/Stranger) and Trial Type (Match/Mismatch). The analysis revealed that the data were 1.23 times more likely under a model with the main effect of Trial Type (BF_10_=1.21, see Fig. 2, Panel A); all other models performed worse than the null. In fact, looking at the critical factor of interest (Identity) for match trials only where self-prioritization typically manifests (Sui et al., 2012), the data were 3.59 times more likely under the null model (BF_10_=.28). Collectively, the results of these analyses speak against a model containing the factor of Identity and suggest that the critical factor of interest is not playing a role in the present pattern of data. That is, contrary to previous self-prioritization results (e.g., Sui et al., 2012) and our hypotheses, the data indicate that participants identified each shape with similar speed.

#### Color estimation judgment

We tested color judgments on models specifying the factors of Cue Identity (Self/Mother/Stranger) and Target Identity (Self/Mother/Stranger). Note that “cue” here refers to which shape’s label was queried in the middle the trial, and “target” refers to which shape’s color was tested at the end of the trial

##### Mean absolute deviation

For models specifying the factors above, data were shown to be 3.127e +26 times more likely under the full model with two main effects and an interaction as compared to the null (BF_10_=3.127e +26). Analysis of the effects for matched models revealed that the strength of this model was primarily attributable to the interaction (BF_Interaction_ = 1.398e+29) with the evidence against the inclusion of Cue Identity (BF_Cue Identity_ = 0.048) and Target Identity (BF_Target Identity_ = 0.046). We followed up this interaction by comparing Cue for all Target Identities. This follow-up analysis revealed participants were more accurate when responding to cued targets (see Table 1 and Fig. 2, Panel B) relative to uncued targets.

**Table 1:** Cuing effects on mean absolute deviation. BF_10_ of Cued/Uncued comparison in brackets. Cued comparison is the value against which the targets (Uncued) were compared. Thus, Self under “Cued comparison” refers to Cue-Self Target-Self, which would be compared against Cue-Self Target-Mother and Cue-Self Target-Stranger. The cueing effect is calculated as a benefit; as such, a positive value demonstrates how much further the uncued target deviated from the cued target

Cued comparison	Target	Cueing effect	BF_10_
Self	Mother	25.50	4,168
	Stranger	26.44	9,425
Mother	Self	23.02	1,885
	Stranger	26.59	103,684
Stranger	Self	24.97	31,519
	Mother	23.83	8,877

##### Memory standard deviation

The data were shown to be 10.99 times more likely under the null hypothesis relative to the next best performing model (a main effect of Cue Identity, BF_10_=0.09, see Fig. 2, Panel C).

##### P(Mem)

For models specifying the factors above, the data were shown to be 2.408e +21 times more likely under a model containing an interaction term relative to the null model. As with Mean Absolute Deviation, analysis of the effects for matched models revealed that the strength of this model was primarily attributable to the interaction (BF_Interaction_ = 9.444e+23) with the evidence against the inclusion of Cue Identity (BF_Cue Identity_ = 0.050) and Target Identity (BF_Target Identity_ = 0.052). Similar to Mean Absolute Deviation, *post hoc* tests revealed that the source of this interaction was attributable to a greater likelihood that the cued stimuli were in memory at the time of the probe as compared to uncued stimuli (see Table 2 and Fig. 2, Panel D).

**Table 2:** Cuing effects on P(Mem). BF_10_ of Cued/Uncued comparison in brackets. Cued comparison is the value against which the targets (Uncued) were compared. Thus, Self under “Cued comparison” refers to Cue-Self Target-Self which would be compared against Cue-Self Target-Mother and Cue-Self Target-Stranger. The cueing effect is calculated as a benefit; as such, a positive value demonstrates how much more likely the cued stimulus was in memory at the time of the probe relative to the uncued stimulus

Cued comparison	Target	Cueing effect	BF_10_
Self	Mother	.31	10,042
	Stranger	.31	29,698
Mother	Self	.28	4,462
	Stranger	.33	9,701
Stranger	Self	.28	2,051
	Mother	.30	7,165

Overall, Experiment 1 did not provide any support for the previously demonstrated self-prioritization in identification processes, the hypothesized effects of self-prioritization on the probability that the stimulus was in memory, or the precision of the memory. Nevertheless, cued stimuli (relative to uncued stimuli) were evidently more likely to be in memory at the time of the probe and were more likely to be remembered more accurately. Confirmation of the cueing effect provides evidence that the measures are sensitive to the processes that we intended to probe within the present series of experiments.

## Experiment 2

Although there was no evidence for self-prioritization in working memory in Experiment 1, it is possible that the high cognitive load of remembering three object locations and their color interferes with processes supporting self-prioritization in cognition, which would prevent any information processing benefits from influencing working memory processes. Indeed, previously it has been shown that divided attention abolishes the self-referential effect in memory (Turk et al., 2013). In Experiment 2, we adapted the task to reduce the cognitive load to examine if self-referential benefits can be observed in working memory.

### Method

#### Participants

As with Experiment 1, a minimum target of 20 participants was set and all participants who signed up completed the experiment. In total, 30 undergraduates enrolled in a first-year psychology course at the University of Toronto participated in this experiment. Variation in the availability of students accounts for the different sample size. All participants were compensated with course credit and provided informed consent before participating. The methods employed were approved by the Office of Research Ethics at the University of Toronto.

#### Stimuli and apparatus

Stimuli and apparatus were identical to Experiment 1.

#### Procedure

Experimental procedures were identical to Experiment 1 with the following exception: during experimental trials only one shape was presented. Following the delay, a label was presented in the same position as the labels had been presented in Experiment 1 – participants were required to respond with the left mouse button if the label was correct and the right mouse button if the label was incorrect. The label remained on screen until a response was made.

#### Data analysis

Data were analyzed in the same way as Experiment 1. Two participants were removed prior to analysis because they failed to reach the 85% accuracy threshold. Henceforth, the final sample in Experiment 2 was n = 28.

### Results and discussion

The aim of the present experiments was to see if self-prioritization creates a higher fidelity representation of self-related objects relative to objects not related to the self. In the first experiment we could not determine whether self-prioritization propagates a higher fidelity representation because we were not able to detect self-prioritization. Nevertheless, we were able to confirm that the dependent measures derived from the color estimation task were sensitive to differences in attentional prioritization by way of cuing. Thus, in Experiment 2, we analyzed the response times of the shape classification judgment to confirm the presence of self-prioritization followed by analyzing the three variables from the color estimation task that probe the probability that the color of the stimulus was in memory and the precision of that representation.

#### Shape classification judgment

Participants were generally accurate in response to the Shape Classification probe (*M* = 97.51%, *SD* = 2.75%); as such, we restrict our analyses to response time. We tested models specifying the factors of Identity (Self/Mother/Stranger) and Trial Type (Match/Mismatch). The analysis revealed that the data were 18,738 times more likely under a model with two main effects and an interaction. Because Match and Mismatch trials generally produce different patterns of results (Sui et al., 2012), we followed up this omnibus analysis by comparing the levels of Identity separately for each type of trial.

##### Match trials

**Fig. 3:**
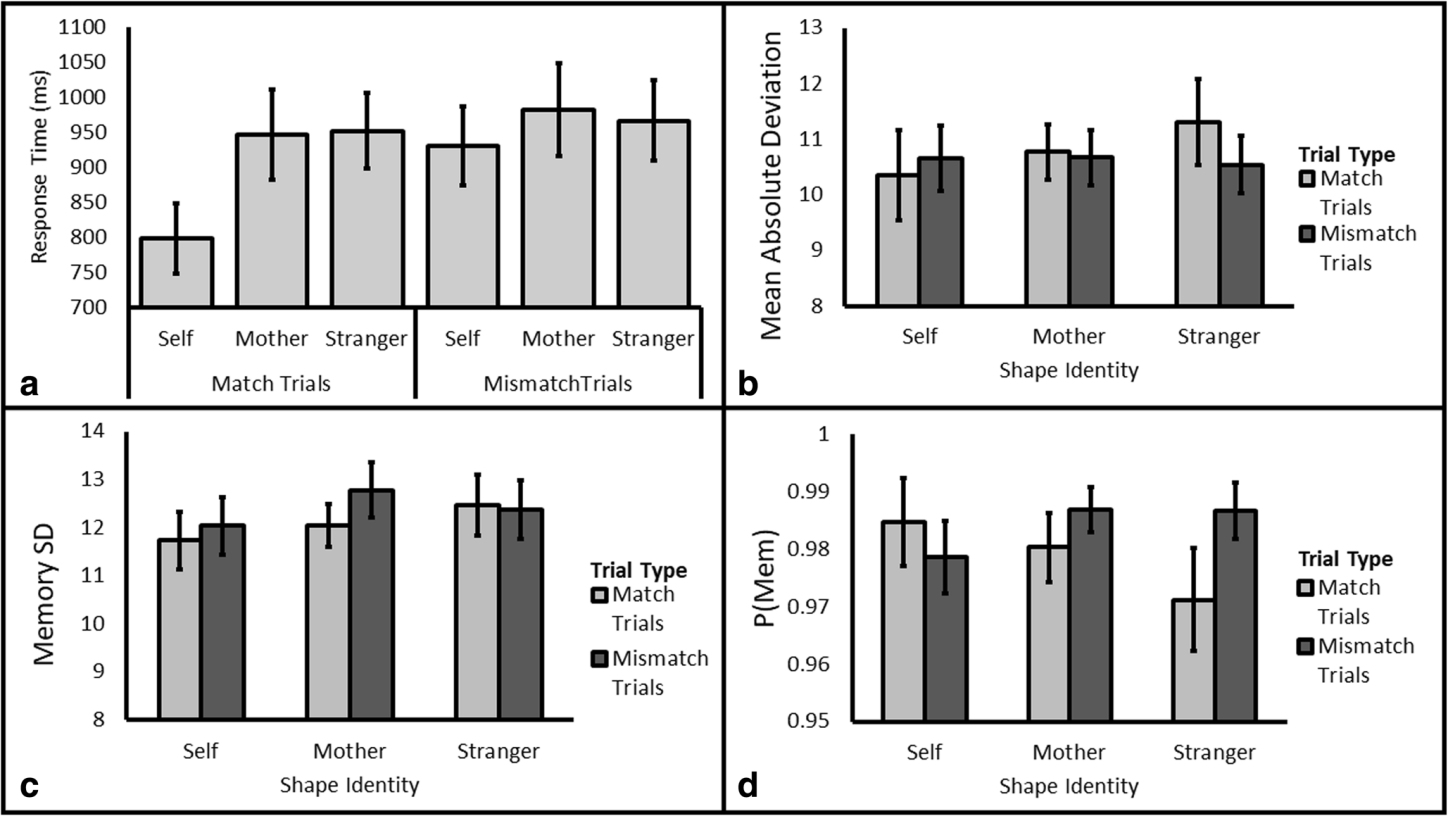
**Panel A:** Response time for match and mismatch trials by Target Identity for Experiment 2. **Remaining panels:** Mean Absolute Deviation (**B**), Memory Standard Deviation (**C**) and P(Mem) (**D**) for Self, Mother, and Stranger shapes by Cue Identity (Self, Mother, and Stranger) for Experiment 2. All error bars are standard error of the mean

Given the *a priori* prediction that self-prioritization would be evident in the data, we tested the prediction that the Self Trials would have shorter response times than the Mother and Stranger Trials. The data were more likely under the alternative hypothesis than the null hypothesis for both of these predictions, BF_10_ = 31.20 and BF_10_ = 73.20, respectively. This result provides very strong evidence for self-prioritization in relation to the Mother stimuli (Self: 798 ms, Mother: 946 ms) and Stranger stimuli (952 ms, see Fig. 3, Panel A). When testing for any difference between Mother and Stranger Stimuli, the data were 4.92 times more likely under the null hypothesis, BF_10_=0.23, which equates to moderate support for no difference between these two stimuli. Overall, these results reveal a clear self-prioritization effect in working memory in match trials that replicates previous work demonstrating the effect when the object and label are simultaneously present (e.g., Sui et al., 2012).

##### Mismatch trials

As with the analysis for Experiment 1, we systematically tested for differences between the levels of Identity. The data were 9.46 times more likely under the alternative hypothesis that self-trials would be faster than mother trials (Self: 931 ms, Mother: 982 ms). The data were only anecdotally more likely under the null for the Self versus Stranger comparison (Stranger: 970 ms, BF_10_=0.90). When testing the hypothesis that there would be no difference between Mother and Stranger Trials, moderate evidence was obtained (BF_10_=4.53).

#### Color estimation judgment

##### Mean absolute deviation

As with response time, we tested models specifying the factors of Identity (Self/Mother/Stranger) and Trial Type (Match/Mismatch). The data were shown to be 4.90 times more likely under the null hypothesis (see Fig. 3, Panel B) relative to the next best performing model (a main effect of Trial Type, BF_10_=0.20). This outcome represents moderate evidence in favor of the null hypothesis.

##### Memory standard deviation

Again, we tested models specifying the factors of Identity (Self/Mother/Stranger) and Trial Type (Match/Mismatch). The data were shown to be 3.48 times more likely under the null hypothesis (see Fig. 3, Panel C) relative to the next best performing model (a main effect of Trial Type, BF_10_=0.29). Again, this outcome represents moderate evidence in favor of the null hypothesis.

##### P(Mem)

Last, we tested models specifying the factors of Identity (Self/Mother/Stranger) and Trial Type (Match/Mismatch) for the measure P(Mem). The data were shown to be 2.56 times more likely under the null hypothesis (see Fig. 3, Panel D) relative to the next best performing model (a main effect of Trial Type, BF_10_=0.33).

Given the presence of self-prioritization in the response times, the absence of effects of an object’s self-relatedness on color memory is striking. We should note that the support for the null model obtained still leaves room to doubt the null hypothesis. Nevertheless, we contend that if self-representational stimuli act to produce attentional prioritization as originally hypothesized then we should observe higher fidelity representations within memory much the same way as location-based cues provided higher fidelity representations in Experiment 1 for which the evidence was extreme. Thus far we have found no support for our hypothesis that the fidelity of the self-stimulus in memory is enhanced relative to other relevant stimuli, and our data are most consistent with the self-recognition hypothesis whereby self-prioritization is restricted to recognizing that a self-representational stimulus is present without any subsequent benefits to encoding in working memory.

## Experiment 3

Although Experiment 2 showed that self-prioritization was evident in response times during retrieval of the target stimulus (Shape Classification), there was little evidence that self-prioritization processes had any impact on the fidelity of the representation in memory or the probability that the stimulus was in memory (Color Estimation). It is possible, however, that performing the initial identity judgment while the object is encoded may enhance encoding and thus the fidelity of the representation in memory. To examine this possibility, we had participants perform a shape classification task while the object was still on screen.

### Method

#### Participants

As with the previous experiments, a target of 20 participants was set and all participants who signed up completed the experiment. In total, 21 undergraduate students who were enrolled in a first-year psychology course at the University of Toronto participated in this experiment. Variation in the availability of students accounts for the different sample size. All were compensated with course credit and provided informed consent before participating. The methods employed were approved by the Office of Research Ethics at the University of Toronto.

#### Stimuli and apparatus

Stimuli and apparatus were the same as previous experiments.

#### Procedure

The experimental task was the same as that used in Experiment 2 except that the label appeared 2.4° below the shape’s center at the same time as the shape. The shape remained on screen until this response. After this, the experiment trial proceeded exactly the same way as Experiment 2.

#### Data analysis

Data analysis was conducted in the same way as previous experiments. Because one participant’s data did not meet the accuracy threshold of 85% their data were removed; thus, n = 20.

### Results and discussion

In Experiment 2, we detected a self-prioritization effect in shape identity verification times, but we still did not detect the hypothesized benefits to memory fidelity. Nevertheless, the conclusion that self-prioritization does not result in the theoretically-predicted enhanced memory representation may still be premature. In Experiment 3, participants completed the color estimation task directly after observing the shape and color. As with Experiment 2, we first analyzed the response times from the shape classification judgment to determine if self-prioritization was present. We then analyzed the three dependent measures derived from the color estimation task to determine if there were any benefits for the self-stimulus in the probability and precision measures for memory. If we are still unable to detect a benefit in the color estimation measures, then our results will be most consistent with the self-recognition hypothesis that self-stimuli have faster access to semantic structures within memory (rapid identification) without further benefits to fidelity of the representation within memory.

#### Shape classification judgment

Participants were generally accurate in response to the Shape Classification probe (*M* = 97.35, *SD* = 2.07); as such we restrict our analyses to response time. We tested models specifying the factors of Identity (Self/Mother/Stranger) and Trial Type (Match/Mismatch). The analysis revealed that the data were 7.72 times more likely under a model with two main effects (Identity and Trial Type) and an interaction as compared to the null model, which was also the next best performing model. Because Match and Mismatch trials typically produce different patterns of results with regards to Identity (Sui et al., 2012), we followed up this omnibus analysis by comparing the levels of Identity separately for each type of trial.

##### Match trials

**Fig. 4:**
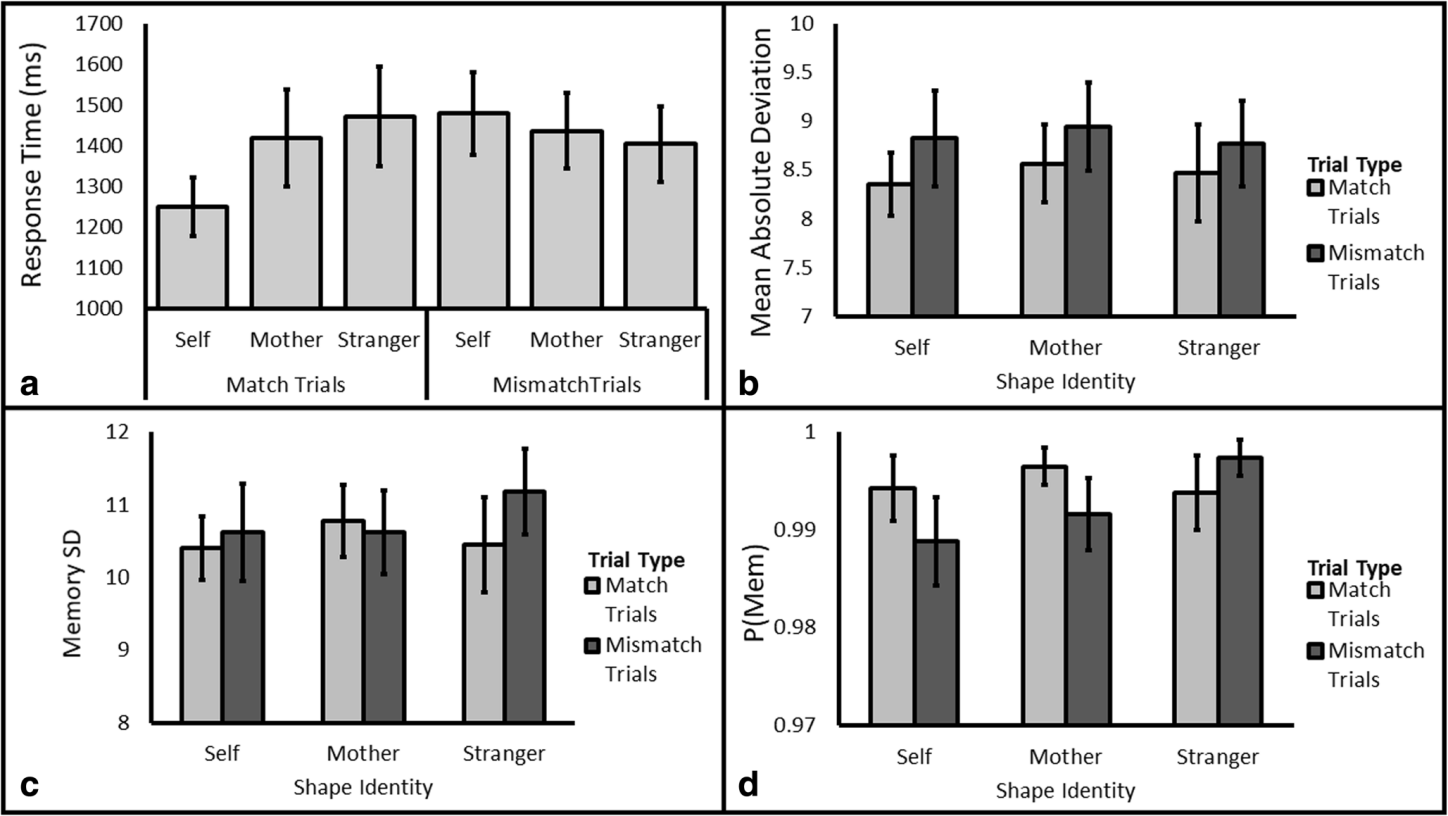
**Panel A:** Response time for match and mismatch trials by Target Identity for Experiment 3. **Remaining panels:** Mean Absolute Deviation (**B**), Memory Standard Deviation (**C**) and P(Mem) (**D**) for Self, Mother, and Stranger shapes by Cue Identity (Self, Mother, and Stranger) for Experiment 3. All error bars are standard error of the mean

Given the *a priori* prediction that self-prioritization would be evident in the data, we tested the prediction that the Self trials would have shorter response times than the Stranger and Mother Trials. The data were more likely under the alternative hypothesis than the null hypothesis for both of these predictions, BF_10_ = 21.63 and BF_10_ = 3.02. This result provides strong evidence for self-prioritization in relation to the Stranger stimulus (Self: 1,250 ms, Stranger: 1,472 ms, see Fig. 4, Panel A) and moderate evidence in relation to the mother stimulus (1,419 ms). When testing for a difference between Mother and Stranger Stimuli the data were 3.29 times more likely under the null hypothesis, BF_10_=0.31, which equates to moderate support for no difference between these two stimuli. Overall, the results are consistent with our hypothesis that self-prioritization would be observed in response times for match trials.

##### Mismatch trials

To follow up on mismatch trials, we systematically tested for differences between the levels of Identity. The data were moderately more likely under the null for both Self versus Mother (Self: 1,479 ms, Mother: 1,437 ms, BF_10_=0.32) and Mother versus Stranger (Stranger: 1,404 ms, BF_10_=0.30) comparisons. Only anecdotal evidence in favor of the null hypothesis was obtained for the Self versus Stranger comparison (BF_10_=0.90). Overall, these results suggest that there were no robust differences between Stimuli for Mismatch trials and are consistent with the previous work that employs the self-association matching task.

#### Color estimation judgment

##### Mean absolute deviation

As with response time, we tested models specifying the factors of Identity (Self/Mother/Stranger) and Trial Type (Match/Mismatch). The data were shown to be 1.002 times more likely under the null model (see Fig. 4, Panel B) relative to the next best performing model (a main effect of Trial Type, BF_10_=0.998). Because the models perform equally well, comparing the null against the next best performing model concerning our factor of interest is warranted. In this case the data were 10.00 times more likely under the null model than a model comprised of the main effects of Identity and Trial Type (BF_10_= .10).

##### Memory standard deviation

Again, we tested models specifying the factors of Identity (Self/Mother/Stranger) and Trial Type (Match/Mismatch). The data were shown to be 3.39 times more likely under the null hypothesis (see Fig. 4, Panel C) relative to the next best performing model (a main effect of Trial Type, BF_10_=.30). Again, this result represents moderate evidence in favor of the null hypothesis.

##### P(Mem)

Last, we tested models specifying the factors of Identity (Self/Mother/Stranger) and Trial Type (Match/Mismatch) for the measure P(Mem). The data were shown to be 3.65 times more likely under the null hypothesis (see Fig. 4, Panel D) relative to the next best performing model (a main effect of Trial Type, BF_10_=0.27). Again, this result represents moderate evidence in favour of the null hypothesis.

Replicating Experiment 2, evidence of self-prioritization was obtained but there was no evidence that this initial response time effect propagated enhanced representations in memory even when the color probe occurred directly after observing the color (as indicated by the null effect in all three color estimation measures). This result is most consistent with the self-recognition hypothesis that self-prioritization is restricted to processes that support the rapid identification of self-stimuli over other stimuli.

## General discussion

The present series of experiments aimed to establish whether self-predilections (1) extend from rapid stimulus processing while the stimulus is visible to speeded judgments about the same stimulus stored in short-term memory, and (2) propagate enhanced encoding for the features of a stimulus thus creating a higher fidelity representation of the stimulus within short-term memory. We contrasted two hypotheses: the visual attention bias hypothesis, which is founded upon the idea that self-stimuli receive greater attentional allocation and, as such, should be encoded with greater fidelity; and the self-recognition hypothesis, which suggests that self-prioritization is restricted to identification-based processes and, as such, has faster access to semantic structures without further benefits regarding the fidelity of the representation. After confirming that color memory differences due to attention were measurable in Experiment 1, Experiments 2 and 3 tested the two hypotheses under reduced cognitive load. The response time data are most consistent with self-prioritization, replicating previous work (e.g., Sui et al., 2012). These self-prioritization effects occur regardless of whether the judgment is done in the context of working memory (Experiment 2) or done while all stimuli are perceptually available (Experiment 3). Despite the emergence of a self-prioritization effect, in both cases there was no effect of stimulus identity on the fidelity of the representation in memory. We contend that if self-representational stimuli act to produce attentional effects then we should have observed higher fidelity representations within memory much the same way as location-based cues provided higher fidelity representations in Experiment 1 for which there was robust evidence.

Thus, our results are most consistent with the self-recognition hypothesis because self-prioritization was only observed with regard to shape classification and not color estimation.

We should note that the moderate support obtained in Experiments 2 and 3 for the null model (no effect of any of our factors) relative to a model containing a main effect of Trial Type regarding the color estimation tasks leaves substantial room for doubt. Indeed, for some measures, the probablity of the data occuring under a null model or a model containing a main effect of Trial Type was almost identical. Nevertheless, we were primarily concerned with stimulus identity. It is very clear that a model containing identity was not a good candidate to explain the presented color estimation data.

There are a few things worth noting about Experiment 1. First, this experiment required that participants retain the location and color of three identity stimuli in working memory. No self-prioritization was observed in either the shape classification judgment or the color estimation task. The lack of effect under high cognitive load is consistent with previous work suggesting that self-based effects are not automatic and require sufficient cognitive resources (Turk et al., 2013). Nevertheless, for the color estimation task participants were more accurate if the shape was cued in the shape classification probe than when it was not cued. This cuing modulation of responses to color estimation confirms that such measures are sensitive to whether or not an object is attended, which was critical for our theoretical rationale.

The disruption of self-prioritization during retrieval (Shape Classification) in Experiment 1 is interesting in the context of the present theory concerning the SAN (Humphreys & Sui, 2015). If one assumes that bottom-up orienting processes interact with a chronically activated self-schema to preferentially tune information processing to self-stimuli, then the self should act in a manner akin to a cueing benefit. That is, if bottom-up orienting processes were engaged in the present experiment, then the self-representational stimuli would have been preferentially attended to regardless of the number of other stimuli present, and thus encoded and retrieved more efficiently. Similarly, there was no fidelity boost to self-stimuli in any experiment, which would be expected if the self-stimulus was allocated greater attentional processing (Zhang & Luck, 2008). Rather, it seems that when participants must remember the color and location of all stimuli present, attention is not allocated preferentially on the basis of stimulus identity. This outcome clearly contradicts a bottom-up orienting account. These results join a growing body of literature that questions the automaticity of possible perceptual and attentional self-referential enhancements (Alexopoulos, Muller, Ric, & Marendaz, 2012; Constable et al., 2019; Golubickis, Falben, Cunningham, & Macrae, 2018). Indeed, the initial allocation of attention (Bundesen, Kyllingsbaek, Houmann, & Jensen, 1997; Gronau, Cohen, & Ben-Shakhar, 2003; Harris, Pashler, & Coburn, 2004; Keyes & Dlugokencka, 2014) and sufficient attentional resources (Alexopoulos et al., 2012; Turk et al., 2013) must be present to elicit a number of self-referential effects.

Even when cognitive load was reduced in Experiments 2 and 3, results still favored the self-recognition hypothesis. The results of Experiment 2 demonstrated rapid self processing even when the stimulus in question was represented in working memory. The results of Experiment 3 replicated the rapid identification of the self-stimulus while it was still visible. Neither of these experiments confirmed our initial hypothesis that self-prioritization should lead to a higher fidelity representation of the self-stimulus within short-term memory. Therefore, the results are most consistent with the self-recognition account whereby self-prioritization terminates with the recognition of a self-based stimulus without further processing benefits. It is possible, however, that such a memory-enhancement could be found if color encoding were somehow made more challenging than in Experiments 2 and 3, but not as challenging as in Experiment 1. However, if such a nuanced balance was required, that would call into question the ecological validity of such an effect.

Overall, the present results suggest that self-prioritization is restricted to features that define selfhood (in this case shape) and not incidental features that co-occur with self-representational features. The extent to which the self-recognition theory might be generalizable to naturally occurring stimuli would be an interesting avenue for future research, and may further inform the way that self is represented within memory. For example, humans may have better memory for self-representational features that do not typically or frequently change, like eye color or the shape of a face, as well as features that might be meaningful (e.g., location), but no preferential memory for things that frequently change, like clothing, that are perhaps less meaningful. Indeed, short-term memory encoding can be restricted to specific object features deemed necessary (Woodman & Vogel, 2008; Chen, Swan, & Wybel, 2016). However, in the task used in the present experiments, both features were task-relevant, and so both were certainly encoded. Overall, it may be simpler to suppose that perceptual processing is not better for self-stimuli, but that it is the association between particular visual features (i.e., shapes) and self-concepts (i.e., names) in long-term memory that is stronger, allowing for quicker decisions. This notion is consistent with theory stemming from the self-reference effect in memory, which states that the self is not special in and of itself other than it provides scaffolding to strengthen semantic memory structures (Symons & Johnson, 1997).

In conclusion, our results suggest that the present self-prioritization effects are more consistent with an improvement in the ability to recognize that a self-representational stimulus is present, without a tendency to devote more perceptual and short-term memory resources to such objects. Given that our results showed that such benefits occurred only for cued stimuli, we suggest that it is more likely that differences in the fidelity of stimulus representations come from general expectations of importance. It is possible that contextual factors that co-occur with self-representational or self-relevant stimuli could modulate such expectations of importance in a manner akin to cued stimuli and thus result in similar enhancements to the detail of the representation in memory. Nevertheless, it is unlikely that self-representational stimuli are perceived and remembered with more perceptual detail merely by virtue of their being self-representational.
